# Assessing the Relationship between Autonomy Support and Student Group Cohesion across Ibero-American Countries

**DOI:** 10.3390/ijerph17113981

**Published:** 2020-06-04

**Authors:** Juan Antonio Moreno-Murcia, Elisa Huéscar Hernández, Luís Cid, Diogo Monteiro, Filipe Rodrigues, Diogo Teixeira, Jeanette M. López-Walle, Argenis Vergara-Torres, José Tristan, Gabriel Gastélum-Cuadras, Julio Cesar Guedea Delgado, Juan Luis Soto Peña, Iván Rentería, Rodrigo Vargas Vitoria, Aquiles Alejandro Almonacid Fierro, Alfonso Valero-Valenzuela, Jorge Flandez, Rudy José Nodari Júnior, Gracielle Fin, Mauricio Rocha Calomeni, Divaldo Martins de Souza, César Augusto de Souza Santos

**Affiliations:** 1 Miguel Hernández University of Elche, Avda. Universidad, s/n, Elche, 30202 Alicante, Spain; ehuescar@umh.es; 2 Sport Sciences School of Rio Maior (ESDRM—IPSantarém), Av. Dr. Mário Soares n-110, 2040-413 Rio Maior, Portugal; luiscid@esdrm.ipsantarem.pt (L.C.); diogomonteiro@esdrm.ipsantarem.pt (D.M.); frodrigues@esdrm.ipsantarem.pt (F.R.); 3 Research Center in Sports Sciences, Health Sciences and Human Development (Portugal), Quinta de Prados, Edifício Ciências de Desporto, 5001-801 Vila Real, Portugal; 4 Life Quality Research Center (CIEQV), Av Dr. Mário Soares n-110, Rio Maior, 2040-413 Santarém, Portugal; 5 Research Center in Sport, Health and Human Development, Lusófona University, Lisbon (Portugal), Campo Grande, 376, 1749-024 Lisboa, Portugal; diogo.sts.teixeira@gmail.com; 6 Sport Organization School, Universidad Autónoma de Nuevo León (México), Cd. Universitaria, s/n, C.P. 66455 San Nicolás de los Garza, Mexico; jeanette.lopezwl@uanl.edu.mx (J.M.L.-W.); argenis.vergaratr@uanl.edu.mx (A.V.-T.); jose.tristanrr@uanl.edu.mx (J.T.); 7 Facultad de Ciencias de la Cultura Física, Universidad Autónoma de Chihuahua (México), C. Escorza, 900, Col. Centro 31000 Chihuahua, Mexico; gastelum@uach.mx (G.G.-C.); jcguedea@uach.mx (J.C.G.D.); 8 Facultad de Educación Física y Deporte, Universidad Autónoma de Sinaloa (México), Av. de las Américas y Prol, Josefa Ortíz de Domínguez s/n, Circuito interior sur-oriente, Ciudad Universitaria, C.P. 80040 Culiacán Rosales, Mexico; jlsoto@uas.edu.mx; 9 Facultad de Deportes, Campus Ensenada, Universidad Autónoma de Baja California (México), Boulevard Zertuche y Boulevard Los Lagos Sin Número, Fraccionamiento Valle Dorado, C.P. 22890 Ensenada, Mexico; irenteria@uabc.edu.mx; 10 Departmento de Ciencias de la Actividad Física, Universidad Católica del Maule (Chile), Avda. San Miguel, Talca, 3605 Maule, Chile; rvargas@ucm.cl (R.V.V.); aalmonacid@ucm.cl (A.A.A.F.); 11 Departmento Actividad Física y Deporte, Universidad de Murcia (España), C/ Argentina, 19, 30720 San Javier, Spain; avalero@um.es; 12 Instituto Ciencias de la Educación, Escuela Pedagogía en Educación Física, Facultad Filosofía y Humanidades, Universidad Austral de Chile (Chile), Independencia, 631 Valdivia, 5090000 Los Ríos, Chile; jflandezv@gmail.com; 13 Department of Physical Education, Universidade do Oeste de Santa Catarina (Brasil), R. Getúlio Vargas, 2125, Flor da Serra, Joaçaba 89600-000, Brazil; rudynodari.junior@unoesc.edu.br (R.J.N.J.); gracielle.fin@unoesc.edu.br (G.F.); 14 Departamento de Educação Física, Laboratório de Biociências da Motricidade Humana do ISECENSA, (Brasil), R. Benta Pereira- Centro, Campos dos Goytacazes 28030-260, Brazil; mauriciocalomeni@gmail.com; 15 Departamento de Ciências do Movimento Humano, Universidade do Estado do Pará (Brasil), Travessa Angustura, 2219, Pedreira, Belém 66087-310, Brazil; divaldodesouza21@gmail.com (D.M.d.S.); cesylamazon@gmail.com (C.A.d.S.S.)

**Keywords:** cross-cultural, self-determination theory, motivational styles, social cohesion, students

## Abstract

Teacher-endorsed supporting behaviors present themselves as key influencers of student adaptive academic and social functions. The objective of this paper was twofold. First, this study sought to test a model in which student-perceived autonomy support was associated with group cohesion, considering the mediating role of basic psychological needs satisfaction and intrinsic motivation. Second, the current study examined the dimensionality of the model across five Western countries, namely Spain, Portugal, Brazil, Chile, and Mexico. A convenience sample of 3033 college students (*M*_age_ = 21.51 ± SD = 3.71) were recruited for the analysis. The results revealed that perceived autonomy support was positively associated with needs satisfaction, being consequently associated with intrinsic motivation and, ultimately, with group cohesion. Additionally, a multigroup analysis revealed that the model was invariant across college students from the different countries. The current results are discussed around the promotion of teacher uses of autonomy-supportive behaviors fostering adaptive outcomes in students regarding positive social relations and that the cultures of Ibero-American countries are equivalent in this process.

## 1. Introduction

In the last decade, based on Theory of Self-Determination (SDT) [1] research, teachers have been considered as key figures in adaptive results—among others, the positive socialization of students—through their motivational styles [2]. Thus, two types of motivational teaching practices have been determined in the past, namely, the autonomy-supportive style and the controlling style [2]. Motivational teaching style is the tone of interpersonal behaviors that teachers use during interactions with their students. While controlling behaviors involves putting pressure on the student to think, feel, and act in a concrete way [3], autonomy support practices are related to self-determined behaviors and positive outcomes across students [4].

The adoption of a motivational style of autonomy support by the teaching figure has been shown to increase the need for autonomy (i.e., student’s sense of having the ability to decide about their own actions), competence (i.e., student’s sense of being able to achieve their proposed goals), and relatedness (i.e., student’s sense of being connected to others), as stated in previous literature [5]. Contrarily, controlling practices have been associated with an increased frustration of basic psychological needs, leading to maladaptive outcomes for students [6]. Through the satisfaction or frustration of basic psychological needs, it is possible to observe a more and a less self-determined motivation across students, respectively, generating positive results in the first case and maladaptive results in the second one [7,8].

Both of these theoretical constructs have given rise to two different motivational patterns known as the bright side of motivation (positive motivational process) and the dark side of motivation (motivational process that would lead to maladaptive outcomes). Although there is consensus in previous works on the functioning of this motivational process to generate differentiated results, the literature is scare on several social aspects across students. In fact, literature has pointed out the role of social triggers in the satisfaction of basic psychological needs to promote a quality (intrinsic) motivation that would eventually be related to different student outcomes (cognitive, emotional, and behavioral) [9,10,11]; however, many of the consequences of both pathways remain unexplored.

In recent years, the phenomena that have to do with harmonious group relations—that is, the positive development within social relations—are getting special attention among scholars and teachers. Specifically, the “team” factor, although mainly studied in the sport context [12], seems to optimize personal efforts in other contexts, such as education [13], and to be able to generate benefits not only at the personal level but, also, at the group level. In this sense, perceived group cohesion is defined as the individual sense of belonging to a group along with the moral feelings associated with the other group members [14]. In this regard, the concept of belonging and the values of morality would be fundamental for adaptive outcomes among students. Additionally, the feeling of belonging is of a cognitive nature, and it has been associated with the promotion of positive relationships among peers [1]. In the case of the feeling of morality, it is of an affective nature and would have its value in promoting the mobilization of the person in achieving the common goals of the organization or group in which the persons belongs. In this sense, although work in this line is still very scarce, a recent study [15] with a sample of college students has pointed out the importance of positive teaching strategies in student self-regulation strategies as a way of student-perceiving group cohesion generating positive academic results. Thus, the value of this dynamic process that guarantees the permanence of the group together in order to achieve the proposed objectives or to satisfy the affective needs of its members [16] should be valued by teachers in order to optimize positive results in students.

From the results of previous research based on the SDT framework [1], there is support that social factors are very important in generating quality motivation (e.g., intrinsic motivation) to the extent in which needs are satisfied. Some works have shown how autonomy-supportive behaviors increase needs satisfaction by catalyzing positive social functioning, such as commitment or prosocial behaviors [17,18]. With respect to group cohesion, although studies are very limited and virtually inexistent, positive results have been found with self-determined motivation [19]. However, to the best of our knowledge, there is virtually no study to date that has examined and explored the influence of student perception of teacher-endorsed autonomy-supportive behaviors on student group cohesion. Therefore, the aim of this study was to analyze the sequence of support for the perceived autonomy support of the teacher, satisfaction of basic psychological needs, intrinsic motivation, and group cohesion in college students. The hypothesis is based on previous research indicating that support for autonomy will fulfill basic psychological needs by relating positively to intrinsic motivation, ultimately catalyzing a greater perception of group cohesion.

### Cross-Cultural Generalizabiliy and Equivalence

Considering the assumptions of the SDT framework, there are different ways in which behavioral practices can be internalized attending to a culture [1,20,21], suggesting that the need to carry out multinational studies to avoid potential limitations in the development of theories is recently proliferating [22]. Specifically, and based on the recommendations of the STD, the objective of these cross-cultural investigations is to avoid possible biases due to inappropriate cultural generalizations. Thus, for example, while some works have verified the nonvariability of the theoretical constructs of SDT between countries [23], other cross-cultural works have found differences across cultures in non-Western cultures, including differences in countries of Western cultures [24]. In one particular study [25] considering 815 teachers from eight different nations, a mediation analysis showed that teachers in collectivistic nations self-described themselves to be more prone to controlling styles due to their perceptions as being a culturally normative classroom practice.

Therefore, considering the recommendation on the need to incorporate intercultural analyses to check the variability of culture on certain motivational constructs, mainly in SDT research [26], this research set has a second objective. As a secondary aim, this study analyzed the invariance of the proposed model across five Western countries, namely Spain, Portugal, Chile, Mexico, and Brazil. Although some of these countries are in Latin America (Brazil, Chile, and Mexico), these countries share the same linguistic background as Spain and Portugal. The concept of the Ibero-American Community of Nations (CIN) or Western Spanish and Portuguese-speaking nationalities, used in scientific and academic research [23] would identify possible variability or equivalence among the same cultures from the different countries. Given the political efforts that have been adopted in order to identify a common cultural heritage, pointing out the cultural affinities that unite the countries of the CIN [27], as well as previous research regarding the universal assumption of psychological needs in each human being [28,29], it is hypothesized that the proposed model would display equivalence across countries, in which there would be no difference between the countries of Spain, Portugal, Chile, Mexico, and Brazil in the relationship to student perception of autonomy support endorsed by teachers, the satisfaction of basic psychological needs, intrinsic motivation, and group cohesion.

## 2. Materials and Methods

### 2.1. Participants

A convenience sample of 3033 students (2051 female; 982 male) aged between 17 and 63 years (*M* = 21.51 ± SD = 3.71) was recruited for the present study. Participants were college students from five different countries, namely Spain, Portugal, Mexico, Chile, and Brazil. Sample statistics of each group are displayed in Table 1.

### 2.2. Measures

The Autonomy Support Scales for Spanish-speaking students [30] and for Portuguese-speaking students [31] were used to measure student perception of autonomy support. Participants were questioned to express their perception of autonomy supportive behaviors endorsed by teachers (6 items: e.g., “My teacher gives me meaningful proposals and positive feedback”) using a 7-point scale ranging from 1 (not agree at all) to 7 (totally agree).

The Basic Psychological Need in Exercise Scales Spanish version [32] and Portuguese version [33] with item adaptation to the classroom settings were used to measure the experiences of autonomy, competence, and relatedness satisfaction. This scale comprises fifteen items assessing autonomy (5 items: e.g., “I feel that I can complete task in the way that I prefer.”), competence (5 items; e.g., “I believe that I can complete personal challenges.”), and relatedness (5 items; e.g., “I feel connected with the people when I engage in the activities.”) satisfaction where responses were given using a 7-point scale anchored from 1 (totally disagree) to 7 (totally agree). Internal consistency for autonomy, competence, and relatedness satisfaction was 0.76, 0.82 and 0.90, respectively. All needs were regressed into a composite factor using similar procedures reported in previous literature [34].

Intrinsic motivation was measured using four items of the Behavioral Regulation in Sport Questionnaire, with proper adaptation to the physical education context in Spanish-speaking students [32] and in Portuguese-speaking students [35]. Students responded to each item based on how they perceive their intrinsic motivation in school-context activities (e.g., “For the pleasure I feel in expanding my knowledge about topics that interest me.”) using a 7-point scale anchored from 1 (totally disagree) to 7 (totally agree).

Spanish and Portuguese native students responded to the Perceived Cohesion Scale [36] to measure how students perceive their bounding with the social environment, specifically group cohesion. It is composed of 6 items (e.g., “I feel that I belong to this group.”). Agreement with each statement was rated on a 7-point scale anchored from 1 (totally disagree) to 7 (totally agree).

### 2.3. Procedures

The study protocol was approved by the Ethical Committee (registration number: 191007203011) and conducted in accordance with the 1964 Helsinki declaration and its later amendments. Schools were identified by the researchers with links in the universities, and school principals and councils were contacted by phone and/or e-mail informing the purpose of the study and signed approval upon agreement prior to data collection. Afterwards, students were contacted based on the e-mail database provided by the school councils. Students were contacted to participate voluntarily, and informed consent was obtained from each participant individually prior to data collection. Responses given by the students were received during the entire school year of each country during the years of 2018 and 2019. Students completed an anonymous online multisection survey, which took approximately 15 min to complete.

### 2.4. Statistical Analysis

Mean, standard deviation, skewness, kurtosis, and bivariate correlations were calculated for each construct under analysis. Cutoffs for normality were considered based on guidelines [37]; we accepted scores within −2/+2 and −7/+7 for skewness and kurtosis, respectively. In addition, composite reliability was assessed suggesting scores above 0.70 as acceptable [38].

A structural equation modeling procedure to test the hypothesized model was conducted using the Weighted Least Square Mean and Variance adjusted (WLSMV) estimator. The model adequacy was assessed according to the following goodness-of-fit indexes: Comparative Fit Index (CFI), Tucker-Lewis Index (TLI), and the Root Mean Square Error of Approximation (RMSEA) with its respective confidence interval (CI 90%). The weighted root mean residual (WRMR) was not considered, since it is not a well-studied fit statistic and has not behaved as well as expected, since this index could provide misleading results under extremely large sample sizes [39].

For cutoffs, CFI and TLI ≥ 0.90 and RMSEA ≤ 0.08 were considered as acceptable [38,40,41]. The confidence interval at 95% (CI 95%) was considered to measure direct and indirect effects among constructs, accepting significance if the CI did not encompass zero [42].

To test the multigroup analysis, the structural SEM model was initially assessed in each group separately. Afterwards, all groups were imputed at the same time in the model to conduct the invariance analysis. Several levels of invariance assumptions were measured according to Morin, Arens, and Marsh [43], namely configural invariance (i.e., factor structure is the same between groups, and items are associated with the same factors); weak factorial invariance (i.e., factor structure and factor loadings are equal between groups); strong invariance (i.e., item factor structure, factor loadings, and item thresholds are equal between groups); and strict factorial invariance (i.e., item factor structure, factor loadings, item thresholds, and item residuals are equal between groups).

Current research adopted differences in CFI (∆CFI), TLI (∆TLI), and RMSEA (∆RMSEA) to evaluate the structural invariance [44]. Structural invariance was considered to be acceptable when differences were ≤ 0.010 [44]. Analyses were conducted in Mplus 7 [45].

## 3. Results

### 3.1. Preliminary Analysis

Descriptive statistics show that participants rated above-average on all constructs under analysis. Univariate skewness and kurtosis were contained within cutoffs, as seen in Table 2. Composite reliability coefficients were above acceptable, and all bivariate correlations were significant, as theoretically expected: (i) autonomy support was positively and significantly correlated with needs satisfaction, intrinsic motivation, and group cohesion; (ii) needs satisfaction was positively and significantly correlated with intrinsic motivation and group cohesion; and (iii) intrinsic motivation displayed a significant association with group cohesion.

### 3.2. Structural Model, Direct and Indirect Effects

The model in each sample provided acceptable fit to the data (see Table 3). All direct effects were significant, as hypothetically proposed in all samples, namely (i) perceived autonomy support displayed a positive and significant association with needs satisfaction, (ii) needs satisfaction was a positive and significant predictor on intrinsic motivation, and (iii) intrinsic motivation was positively and significantly associated with group cohesion.

Looking at the indirect effects, positive and significant were found in all country samples: (i) perceived autonomy support displayed a significant indirect effect on intrinsic motivation and group cohesion, and (ii) needs satisfaction showed a positive and significant indirect effect on group cohesion. Beta coefficients of direct and indirect effects and their respective CI 95% across samples are shown in Table 4.

### 3.3. Multigroup Analysis

The structural model was invariant between groups, since invariance criteria were respected: namely, configural, weak, strong, and strict factorial. Looking specifically to the differences in CFI, TLI, and RMSEA, they were all below the cutoffs. For detailed information, see Table 5.

## 4. Discussion

The current study aimed to examine the relationships between how college students perceive teacher-endorsed autonomy-supportive behaviors, the satisfaction of basic psychological needs, intrinsic motivation, and group cohesion. The proposed model advances previous research applying the SDT framework by analyzing its effectiveness in accounting for adaptive outcomes such as group cohesion. The present study is expected to provide further robust evidence to inform the development of protocols aimed at promoting crucial academic outcomes delivered in the classroom setting. The results supported the hypothesis and found that perceived autonomy support was positively related to group cohesion, in which needs satisfaction and intrinsic motivation displayed mediating roles. Although the scientific literature has so far supported this theoretical sequence among SDT constructs [46], confirming that it catalyzes positive consequences in the student related to positive social relations such as prosocial behavior [18], to the best of our knowledge, the relationships between perceived behaviors from others and prosocial behaviors among students is still under-researched.

The present study supports several of the key premises of the SDT framework when considering the relationship between motivational constructs and adaptive outcomes. Specifically, perceived autonomy support predicted needs satisfaction in the classroom setting directly. Results provide further evidence that a student’s experience of needs fulfillment is related to their behavior regulation towards a more self-determined (i.e., intrinsic motivation) behavior. Current findings support the central premises of the SDT framework: the positive impact of perceived autonomy-supportive behaviors on needs satisfaction and, consequently, the critical aspect of needs fulfillment as an indicator of internalized behavior and optimal functioning. This finding has crucial important implications for interventions to promote greater levels of autonomous motivation, in which the student considers classroom activities as important, resulting in greater academic success [47]. Previous studies suggest that college teachers can foster intrinsic motivation in the classroom setting by displaying key autonomy-supportive behaviors such as providing evaluative feedback, choice, and clear rationale and that such interpersonal behaviors lead to increased perceptions of autonomy support in college students [4,30,31]. If such interpersonal behaviors are effective in changing the perceptions of autonomy support among students, they could lead to adaptive outcomes related to other social interactions. While the effects of such protocols should be empirically tested, present findings may signpost a key strategy that may have utility in promoting positive changes in needs satisfaction, intrinsic motivation, and, consequently, group cohesion.

Current findings found indirect effects of perceived autonomy-supportive behaviors endorsed by teachers in group cohesion. Indeed, the teacher who adopts a motivational style that supports the autonomy of the student, favoring effort and cooperation towards learning, is also functioning as a social reference model for the student that can contribute to being able to more easily adopt positive social patterns [48]. In SDT research, Balaguer et al. [12] pointed out that, when the figure of authority favors a positive atmosphere, it facilitates the desire between group members within the group to remain cohesive and to maintain close ties. Forthcoming studies should explore more adaptive outcomes using this empirical sequence, which confirms the benefits of autonomy-supportive teaching [17], in order to verify the relationship of cohesion and student academic success, as it has been pointed out in previous literature [49].

Given the exponentially growing interest in motivational models as its implication in adaptive outcomes, it is important to rely on reliable and solid models that provide a robust picture on how teachers can endorse in need-supportive behaviors. The use of autonomy-supportive behaviors seems of the utmost importance, as the understanding of the college student internal frame of reference, in which students perceive a sense of volitional choice, should be provided. In this sense, teachers are encouraged to provide rationale for a requested task, encourage students’ initiation towards a given activity, and express student ownership when they have self-initiation. The results of this study could help to illuminate the directional orientations between interpersonal behaviors and prosocial interactions. All in all, when college students experience need-supportive behaviors from teachers, their needs are satisfied, and they experience a form of motivation that is more autonomous, consequently leading to positive outcomes, as demonstrated in the current study.

### 4.1. Cross-Cultural Analysis

The second aim of the present study was to explore the invariance of the model in samples of college students from different Western countries who shared similar languages (i.e., Spanish and Portuguese). Specifically, this study intended to examine if the proposed model would maintain equivalence across Spain, Portugal, Brazil, Chile, and Mexico. Measurement experts [50] have maintained that multigroup analysis is at the heart of the process of generalization. Overall, evidence has emerged for the invariance of the proposed model across the diverse countries that have been examined. Present results showed that the model had the same theoretical structure for each study group, similar to the existing literature [40].

Current results show that college students speaking different languages did not differ in the level of motivational constructs and group cohesion. This makes sense given the assumptions proposed by Ryan and Deci [1]. Specifically, these results are aligned with the SDT framework in the sense that the theory argues the universal meaning of the three psychological needs regarding their robust effects on the adaptive development and positive functioning of people regardless of the cultural context [1,5]. In this regard, this study considering samples from Spanish and Portuguese-speaking countries indicates that culture may not be more important than the motivational constructs underlying the motivational framework of SDT. Previous research has corroborated with this argument in countries such as the United States, Australia, and Belgium to Mexico, Peru, Malaysia, China, and the Philippines [51,52], agreeing in the case of Mexico with the data of the present study. Regarding the rest of the countries considered in this study, there are no previous works in this line. The fact that the model displayed equivalence across cultures provides strong support of the generalizability of the motivational constructs that are related to previous SDT research.

It is worth mentioning that, regarding the motivational orientations among students, findings of previous research carried out with Western countries [22,52] do not seem to be conclusive, findings only in some cases invariant among samples [23,53]. Therefore, this work could be considered a preliminary study in the analysis of intercultural differences in the motivational processes of Latin American and Iberian students. More work is needed in this sense in order to increase the knowledge on the possible differences in the understanding of the measurement variables used, as well as other variables that may explain the current findings. The main finding of this work was to provide support for an empirical model based on SDT in which the motivational style of autonomy support perceived by college students catalyzes the perceived group cohesion through the satisfaction of basic psychological needs and intrinsic motivations, being equivalent among five countries native to Spanish and Portuguese-speaking cultures.

### 4.2. Strengths, Limitations, and Avenues for Future Research

Strengths of the current study are the adoption of the SDT framework, an appropriate model that provides a clear set of associations on the motivational determinants across five countries, the use of robust, previously validated measures for Spanish and Portuguese native speakers, and the adoption of robust statistical analyses to account for possible variability across countries. Although the current study investigated students with diverse cultural backgrounds, there are some limitations that should be acknowledged. First, the sample consisted of college students. It would be especially interesting to investigate less-educated (e.g., high school and middle school students) students on how they perceive autonomy-supportive environments and how they are related to group cohesion and compare the results with the current findings. Second, the samples were from Spanish and Portuguese native-speaking countries, preventing the generalization of other cultures. Given the interindividual variability in cultural backgrounds [54], future research should explore and analyze the current model in different cultures and populations (e.g., Western and Eastern nationalities). Third, the methodological limitation was that all reported measures were cross-sectional in nature, preventing us from drawing any causal conclusions. Thus, future studies could implement an experimental design involving observational measures where teachers could also express their perceptions of their behaviors. Again, longitudinal manipulations are paramount for measuring inferences. Last, since no hypotheses were proposed considering sociodemographic (e.g., gender and age) and academic course (i.e., type of specialization) variables, more studies are paramount for examining the possible variabilities across moderators.

## 5. Conclusions

The current study contributed to the cross-cultural understanding of a teaching motivational style, finding that autonomy-supportive behaviors contribute to needs satisfaction, intrinsic motivation, and group cohesion in college students from five countries, supporting the previous literature [23,55,56]. Specifically, these results support the strength of the SDT assumptions regarding their intercultural relevance in Spanish and Portuguese native-speaking countries, which encourages further knowledge in subsequent works regarding intercultural variability. The SDT was applied to outline how student perceptions of need-supportive behaviors endorsed by teachers are associated with needs fulfillment, intrinsic motivation, and, ultimately, group cohesion. Furthermore, these relations were equal by cultural differences, displaying how strongly students from different cultural backgrounds value autonomy-supportive behaviors endorsed by teachers. The present findings suggest that the motivational style students perceive from teachers is essential for optimal functioning and adaptive outcomes across college students from all cultural backgrounds.

The present results confirm the overall positive associations between SDT motivational constructs and group cohesion that have been demonstrated in the previous literature. Consequently, manipulating group processes by teacher-induced team-building activities could be a promising avenue for the promotion of continuous group cohesion among college students. The acknowledgement of group dynamics by teachers seems to be a paramount prerequisite when implementing school-based activities in college students. These assumptions seem to fit in Spanish and Portuguese native-speaking students, since the proposed model was equivalent among the countries. Thus, by analyzing group processes that have been developed by students over a period of time, one could propose adaptive outcomes such as greater academic grades [47] and more engagement in leisure-time physical activities [57]. More research is warranted to examine this hypothesis and relate it to the general classroom setting.

## Figures and Tables

**Table 1:** Sample characteristics.

Countries	*N*	F	MIN–MAX	M ± SD
Spanish	602	456	18–48	22.39 ± 1.24
Portuguese	469	335	18–63	21.82 ± 3.90
Mexican	1177	784	18–58	20.81 ± 3.17
Chileans	372	260	18–34	21.33 ± 2.24
Brazilian	413	216	18–54	22.03 ± 3.97

Note: *N* = sample size, F = female, MIN–MAX = age range, and M ± SD = age mean and standard deviation.

**Table 2:** Descriptive statistics, composite reliability, and correlations.

Factors	M	SD	S	K	CR	1	2	3	4
1. PAS	3.94	0.81	−1.35	2.09	0.91	1			
2. BPNS	4.07	0.68	−1.16	2.73	0.70	0.50 **	1		
3. IM	5.74	1.12	−1.00	1.02	0.83	0.18 **	0.27 **	1	
4. GC	5.49	1.40	−1.08	0.89	0.95	0.27 **	0.47 **	0.34 **	1

Note: PAS = perceived autonomy support, BPNS = basic psychological need satisfaction, IM = intrinsic motivation, GC = group cohesion, M = mean, SD = standard deviation, S = skewness; K = kurtosis, and CR = composite reliability; ** *p* ≤ 0.01.

**Table 3:** Model fit indexes.

Model	WLSMVχ2	df	CFI	TLI	RMSEA (90% CI)
Total	5183.550 *	296	0.968	0.965	0.074 [0.072, 0.076]
Spanish	1093.928 *	296	0.976	0.974	0.067 [0.063, 0.071]
Portuguese	1167.984 *	296	0.955	0.951	0.079 [0.075, 0.084]
Mexican	2494.885 *	296	0.969	0.966	0.079 [0.077, 0.082]
Chileans	831.890 *	296	0.979	0.977	0.070 [0.064, 0.075]
Brazilian	1305.062 *	296	0.970	0.967	0.091 [0.086, 0.096]

Note: WLSMVχ2 = Weighted Least Square Mean and Variance Adjusted Chi-Square Test, df = degrees of freedom, CFI = Comparative Fit Index, TLI = Tucker-Lewis Index, RMSEA = Root Mean Squared Error of Approximation, and 90% CI = 90% confidence interval of RSMEA; * *p* < 0.001.

**Table 4:** Direct and indirect effects in all samples.

Path	Total			Spanish			Portuguese			Mexican			Chileans			Brazilian		
	β	CI 95%		β	CI 95%		β	CI 95%		β	CI 95%		β	CI 95%		β	CI 95%	
		Low	Up		Low	Up		Low	Up		Low	Up		Low	Up		Low	Up
Direct Effects																		
PAS → BPNS	0.57	0.53	0.61	0.54	0.44	0.63	0.65	0.56	0.66	0.61	0.56	0.67	0.67	0.57	0.77	0.56	0.47	0.68
BPNS → IM	0.67	0.63	0.72	0.75	0.66	0.84	0.89	0.80	0.97	0.64	0.56	0.71	0.53	0.40	0.65	0.77	0.67	0.87
IM → GC	0.64	0.61	0.68	0.61	0.54	0.69	0.56	0.46	0.74	0.71	0.66	0.76	0.70	0.61	0.78	0.55	0.45	0.65
Indirect Effects																		
PAS → GC	0.25	0.22	0.28	0.25	0.18	0.32	0.32	0.24	0.41	0.28	0.22	0.33	0.25	0.16	0.33	0.24	0.17	0.32
PAS → IM	0.39	0.34	0.43	0.40	0.31	0.50	0.57	0.48	0.67	0.39	0.33	0.45	0.35	0.24	0.46	0.44	0.34	0.55
BPNS → GC	0.43	0.39	0.47	0.46	0.38	0.52	0.50	0.40	0.59	0.45	0.39	0.51	0.37	0.26	0.48	0.43	0.34	0.51

Note: Low = lower, Up = upper, PAS = perceived autonomy support; BPNS = Basic Psychological Needs Satisfaction; IM = Intrinsic Motivation; GC = Group Cohesion; β = standardized coefficient; CI 95% = Confidence Interval at 95%.

**Table 5:** Multigroup analysis of the structural model across all countries.

Model	WLSMVχ2	∆χ2	df	∆df	CFI	∆CFI	TLI	∆TLI	RMSEA	∆RMSEA
Configural Invariance	9355.805 *	-	2021	-	0.961	-	0.969	-	0.077	-
Weak Factorial Invariance	9947.596 *	591.791	2047	26	0.958	0.003	0.967	0.002	0.080	0.003
Strong Invariance	10,008.286 *	652.481	2068	47	0.958	0.003	0.967	0.002	0.080	0.003
Strict Factorial Invariance	9526.751 *	170.946	2064	43	0.960	0.001	0.969	0.000	0.077	0.000

Note: WLSMVχ2 = Weighted Least Square Mean and Variance Adjusted Chi-Square, ∆χ2 = difference in χ2, df = degrees of freedom, ∆df = differences in df, CFI = comparative fit index, ∆CFI = differences in CFI, RMSEA = Root Mean Squared Error of Approximation, and ∆RMSEA = differences in RMSEA; * *p* < 0.001.
